# The Healthy Baby Flipbook: piloting home-based counseling for refugee mothers to improve infant feeding and water, sanitation, and hygiene (WASH) practices

**DOI:** 10.1080/16549716.2018.1560115

**Published:** 2019-01-18

**Authors:** Ahmar Hashmi, Verena I. Carrara, Paw Bay Nyein, Mu Chae Darakamon, Prakaykaew Charunwatthana, Rose McGready

**Affiliations:** a Shoklo Malaria Research Unit, Mahidol-Oxford Tropical Medicine Research Unit, Mahidol University, Mae Sot, Thailand; b Department of Family Medicine, Faculty of Medicine, Chiang Mai University, Chiang Mai, Thailand; c Department of Medicine, Swiss Tropical and Public Health Institute, Basel, Switzerland; d Mahidol-Oxford Tropical Medicine Research Unit, Faculty of Tropical Medicine, Mahidol University, Bangkok, Thailand; e Centre for Tropical Medicine and Global Health, Nuffield Department of Medicine, University of Oxford, Oxford, UK

**Keywords:** Infant nutritional physiological phenomena, breastfeeding, feeding behavior, counseling, vulnerable populations

## Abstract

Problems in growth and undernutrition manifest in early infancy, with suboptimal breastfeeding and inadequate complementary feeding remaining strong risk factors for chronic undernutrition in infants. No published studies exist on educational interventions to improve infant feeding practices among refugees or displaced persons in low and middle-income (LMIC) settings. The objective of this study was to create and pilot educational materials for home-based counseling of refugee mothers along the Thailand–Myanmar border to improve appropriate infant feeding and water, sanitation, and hygiene (WASH) behaviors. Mothers of infants received counseling on appropriate infant feeding and WASH practices on a monthly basis for a total of six months from infant age three months until nine months. Educational materials were designed to feature a basic script for health workers and photos of locally available, appropriate foods. Of the 20 mothers participating in this pilot, infant feeding and WASH behaviors improved within 1 to 2 months of the first visit, including exclusive breastfeeding, minimum acceptable diet, and safe disposal of infant stool. This pilot demonstrates improvement in maternal infant feeding and WASH practices in a small set of refugee mothers, providing evidence for counseling measures to improve infant health in vulnerable populations.

## Background

Recent analyses reveal that problems in growth and undernutrition manifest in early infancy [1–4]. Suboptimal breastfeeding and inadequate complementary feeding remain strong risk factors for chronic undernutrition in infants [1]. There is a growing number of studies demonstrating benefit and impact of counseling and educational interventions to improve breastfeeding and complementary feeding for infant nutrition [5–10]. However, in spite of displaced persons having reached record numbers nearing 68.5 million people globally [11], publications to date have only focused on preserving breastfeeding in crisis situations [12,13]. No published studies exist on educational interventions to improve infant feeding practices among refugees or displaced persons in low and middle-income (LMIC) settings. In addition, there is limited information available on creation of materials for populations with low literacy rates. This study piloted home-based, one-on-one counseling for a largely illiterate population of refugee mothers in Mae La refugee camp in Thailand to validate potentially, effective counseling techniques for improved maternal infant feeding and water, sanitation, and hygiene (WASH) practices. This paper highlights methods and materials used for the counseling intervention and reports the results of the pilot.

## Methods

### Study population

This pilot was conducted with recently delivered women attending the Shoklo Malaria Research Unit (SMRU) clinic in Mae La refugee camp (MLA) from October 2013 to June 2014. The SMRU MLA clinic provides antenatal , obstetric, pediatric, and general medical care to refugees. Refugees are predominantly of Burman or Karen ethnicity who identify as Buddhist or Christian. Muslims constitute a significant minority within the MLA refugee camp. Importantly, nutrition education was performed in a refugee camp where meal rations were provided to this food secure population.

### Study design

Mother–infant pairs from a cohort evaluating the impact of malaria in pregnancy on fetal growth were selected to receive a home-based, one-on-one counseling intervention. Mothers with term, healthy infants aged two months at the time of recruitment were approached in the SMRU MLA clinic and, after giving consent to participate, received monthly household visits from infant age three months onwards for a total of six months. House visits took 30 minutes or less and were conducted by a study nurse previously unknown to the participants. The study nurse would record mothers’ practices through direct questions and/or observations, following World Health Organization (WHO) recommendations and definitions for appropriate infant feeding [14] and additional indicators to assess appropriate WASH practices (Table 1). In addition to recording behaviors of interest according to WHO definitions, monthly surveys collected information on household expenditures, food resources accessed, and food items purchased the week prior. Feeding practices and meal preparation were observed when possible. Each home visit would culminate with the study nurse counseling mothers on appropriate behaviors for feeding, sanitation, and hygiene. Statistical analysis focused on behavior change over time.

**Table 1.** Outcomes of interest for feeding, hygiene, and sanitation behaviors in the 24 hours prior to cross-sectional survey (WHO 2007).

Outcomes of interest	Definition
Exclusive breastfeeding	Infants < 6 months fed breast milk while receiving no other food or liquid, not even water, except for vitamins, mineral supplements, or medicine
Predominant breastfeeding	Infants < 6 months receiving predominantly breast milk and also receiving liquids, but not soft food
Partial breastfeeding	Infants < 6 months receiving breast milk and any soft food
Minimum Dietary diversity	Infant ≥ 6 months fed 4 or more food groups
Meal frequency	Minimum of 2 meals for infants 6–8 months and 3 meals for infants 9–12 months if breastfed; minimum of 4 meals aged 6–12 months if not breastfed
Minimum acceptable diet	Infant ≥ 6 months meeting minimum requirements for dietary diversity and meal frequency
Handwashing	Of mothers preparing meals, those who appropriately washed hands before meal preparation
Safe stool disposal	Of mothers with an improved form of sanitation (latrine, flush, or pour-flush toilet), those that discarded infant stool using an improved form of sanitation
Safe water	Infants ≥ 6 months fed boiled or bottled water

### Educational materials: ‘The Healthy Baby Flipbook’

**Figure 1.**
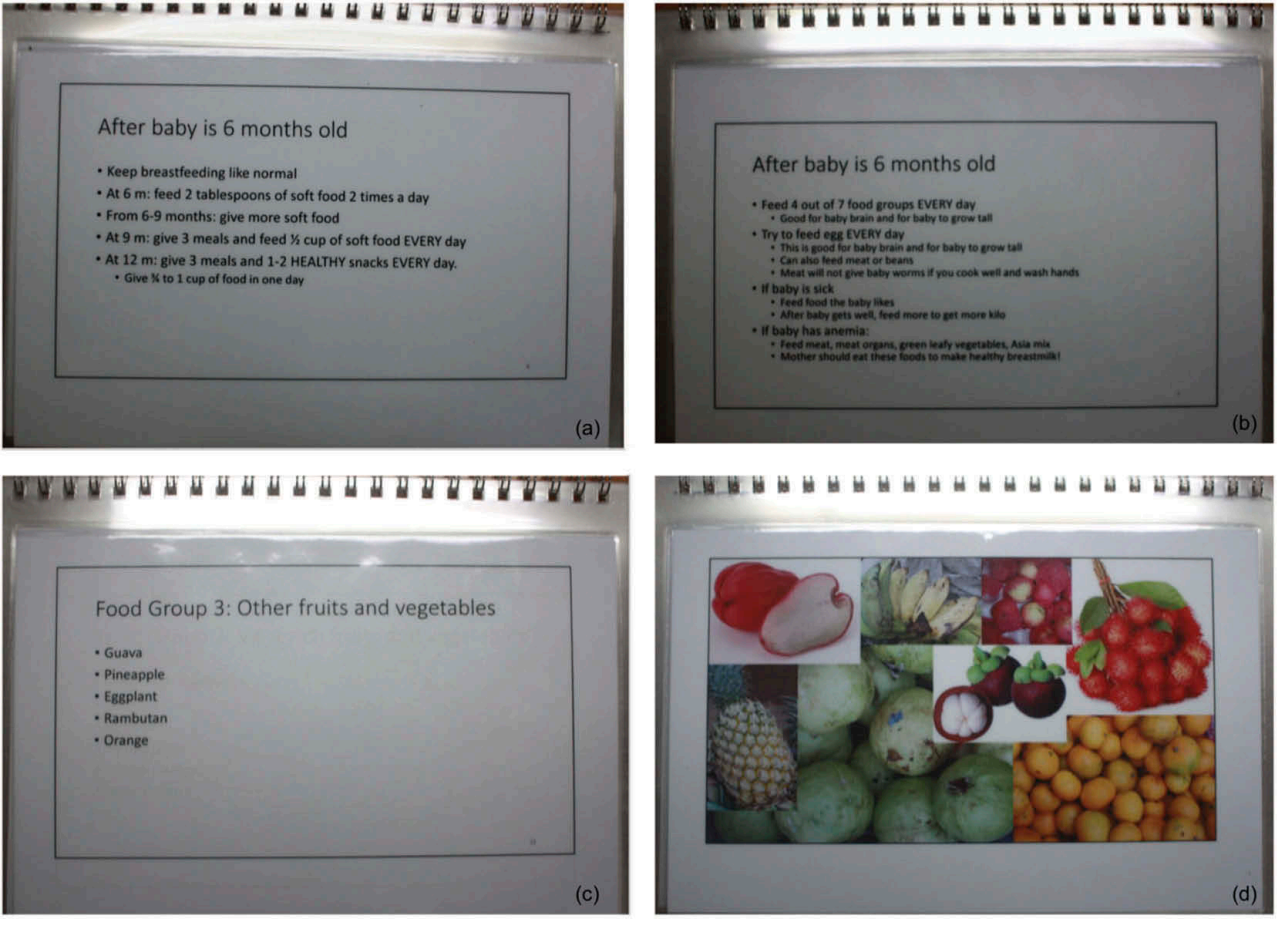
Examples of pages in the “Healthy Baby Flipbook” flipbook. (a) and (b) includes examples of script tailored to behaviors common among refugee mothers; (c) has food items listed to remind the counselor, while the opposite side (d) features photos of locally available, nutritious foods facing the mother receiving counseling.

Researchers (AH, VC) prepared and provided the study nurse with a flipbook featuring a script in basic English and photos of locally available foods acceptable for complementary feeding of the infant after six months of age (Figure 1). Designed for a largely illiterate population, each page of the flipbook was double-sided: one side with the script faced the study nurse while the opposite side was simultaneously visible to the woman and showed photos of locally available foods (Figure 1). The flipbook begins with counseling on WASH behaviors, followed by exclusive breastfeeding for infants of less than six months, then highlights the seven food groups and amounts of breastfeeding and complementary foods according to infant age as outlined by the WHO [14]. Important elements of the script were adapted and updated based on findings from focus group discussions that provided common rationale among refugee mothers for inappropriate behaviors (Figure 1). Although this population may be considered ‘food secure’ with rations providing a large proportion of daily nutritional requirements for households, households supplement remaining nutritional needs according to their financial means. As home-based counseling sessions also collected data on monthly household expenditures for food, the study nurse could tailor counseling for mothers by providing cheaper food options for infants and specific examples of low-cost meals to meet minimum acceptable diet and diet diversity requirements. Training of the study nurse took approximately one week, which included testing the intervention with pregnant women prior to implementation of the pilot. The basic English script allowed the study nurse to develop her own style in delivering the counseling in either Karen or Burmese language. Researchers (AH, MCD) provided supervision and support for counseling over the course of the pilot.

## Results

Out of the 34 mothers with infants eligible for inclusion at the time of recruitment, 20 mothers consented to participate in the longitudinal cohort (59%). A total of 132 household visits were conducted and a median of 7 visits per household (range 3 to 7) between October 2013 and June 2014. One mother lived outside MLA and was interviewed in the clinic at four, six, and nine months. The remaining 19 were followed up monthly in their homes from baseline at age 3 months until the infant reached 9 months of age; 15 (79%) were seen every month with 3 mothers missing only one appointment due to travel to neighboring Myanmar.

The proportion of exclusively breastfed infants increased from 42% (8/19) at 3 months to 65% (11/17) at 5 months. Among mothers who had prepared the family meal the day prior to interview, handwashing was 94% (15/16) at baseline and reached 100% at 6- (13/13) and 9-month (20/20) visits. Infants at 6 months of age were fed inadequately; 5% (1/20) were fed adequate dietary diversity; 10% (2/20) had received the appropriate meal amount, and no infants had consumed a minimum acceptable diet. These proportions increased to 90% (18/20), 100% (20/20), and 90% (18/20), respectively, by the time the infants reached 9 months. Appropriate dietary diversity and minimum acceptable diet increased within 2 months from the 6-month interview from 5% (1/20) to 58% (11/19, p < 0.001) and from 0% (0/20) to 47% (9/19, p < 0.001), respectively. After excluding the one mother living outside MLA without an improved form of sanitation, safe disposal of infant stool improved progressively from 16% (10/19) at 6 months to reach 100% (19/19; p = 0.040) at the 9-month interview.

## Discussion

Given the growing burden of displaced populations globally, this study presents a counseling program conducted by local health workers for improved infant feeding practices in a marginalized, largely illiterate, refugee population [15]. Baseline data from a cross-sectional survey of 103 women with infants between 6 and 12 months from the same original cohort in MLA camp showed poor rates of diet diversity and minimum acceptable diet at 20% and 2%, respectively (Hashmi, unpublished data). It is important to reiterate that this nutrition counseling was conducted in what can be considered a food secure population, but likely representative of displaced persons establishments elsewhere under the purview of the United Nations High Commissioner for Refugees. It is also important to note that this population already has baseline data suggesting high rates of breastfeeding initiation and breastfeeding duration [16], which may also positively impact uptake of behavior change education [8]. This pilot follows recommendations from recent systematic studies summarizing findings for appropriate counseling for improved outcomes in infant feeding – including exclusive breastfeeding, breastfeeding duration, and complementary feeding – in meeting diet diversity and minimum acceptable diet requirements for infants six months of age or older [5–10]. Although the sample size is quite small and inferences as to the significance of these behavior changes are therefore limited, the rapidity with which mothers changed behaviors in this pilot study is promising and provides further evidence that verbal messaging in the home and/or the community can improve infant feeding practices in resource-poor settings with low rates of literacy [10]. Results of this pilot have been communicated with local stakeholders, including health worker staff at SMRU, local organizations engaged in programming for infant feeding in refugee and migrant communities along the border, and the nutrition working group of The Border Consortium – a technical working group bringing together organizations providing rations and working on nutrition across all refugee camps in Thailand. Further research includes studying the impact of this counseling intervention on infant growth and its cost-effectiveness along the Thailand–Myanmar border.

## Conclusions

This pilot demonstrates improvement in maternal infant feeding and WASH practices in a small set of refugee mothers, providing evidence of counseling measures to improve infant health in vulnerable populations.

## Paper context

Suboptimal breastfeeding and complementary feeding remain strong risk factors for chronic infant malnutrition. No published studies exist on educational interventions to improve infant feeding practices among refugees or displaced persons in LMIC settings. This study created and piloted educational materials for counseling of refugee mothers along the Thailand–Myanmar border to improve infant feeding and water, sanitation, and hygiene practices. This pilot provides evidence of counseling measures to improve infant health in vulnerable populations.
